# Do the rates we report misinform the local programs?

**DOI:** 10.12669/pjms.303.4912

**Published:** 2014

**Authors:** Mohammad Darvishi, Atefeh Noori, Shervin Assari, Babak Moazen

**Affiliations:** 1 Mohammad Darvishi, Assistant Professor, Infectious Diseases and Tropical Medicine Research Center (IDTMRC), AJA University of Medical Sciences, Tehran, Iran.; 2 Atefeh Noori, Non-communicable Diseases Research Center, Endocrinology and Metabolism Population Sciences Institute, Tehran University of Medical Sciences, Tehran, Iran.; 3 ShervinAssari, Center for Research on Ethnicity, Culture, and Health (CRECH), Department of Health Behavior and Health Education, University of Michigan School of Public Health, Ann Arbor, USA.; 4 Babak Moazen, Non-communicable Diseases Research Center, Endocrinology and Metabolism Population Sciences Institute, Tehran University of Medical Sciences, Tehran, Iran.

**Keywords:** Communication, Correction, Error, Misinformation, Published Erratum, Research Report

Prevalence of the major infectious diseases such as HIV/AIDS, Tuberculosis, and different types of viral hepatitis is higher among prisoners than in the general populations.^1^ This issue might be justified by the higher frequency of high-risk behaviors such as shared injection, unprotected sex especially among Men who have Sex with Men (MSM) in prisons, different types of skin penetration, as well as unsanitary environment of the prisons in many areas of the world.^2^^-^^6^ Despite high vulnerability of prisoners, the existing data about the prevalence of infectious diseases among them is very limited.^7^ This may be due to limitation in gaining access to this high-risk population.

During the process of data collection for a systematic review, we found an original article entitled: “HIV Infection, HIV/HCV and HIV/HBV co-infections among Jail Inmates of Lahore”, written by Nafees et al., published in Pakistan Journal of Medical Sciences.^8^ We highly acknowledge the researchers to choose this subject, regarding existing limited data access in terms of HIV/AIDS, and viral hepatitis in prisons is highly valuable. However, we observed an error to report the HIV prevalence among male participants. Drawing on the results, there were 94 HIV positive men, out of total 4498 male inmates, which equal 2.08% HIV prevalence rate. But unfortunately it seems that the researchers have calculated the percent of HIV positive male inmates by dividing the number of HIV positive males on the total number of the prisoners (94/4915) and reported the prevalence rate as 1.91%, which is not very accurate.

This should be considered that making mistake is inevitable in research. This is evidenced by the considerable number of the manuscripts, which have been corrected by the authors after publication.^9^^-^^15^ However, since the error reported here may influence the reliability of global estimations, and also misinform the local programs, we respectfully ask the researchers to correct the above-mentioned error by writing an erratum.
